# Exploring the vast potentials and probable limitations of novel and nanostructured implantable drug delivery systems for cancer treatment

**DOI:** 10.17179/excli2023-6747

**Published:** 2024-02-01

**Authors:** Maryam Ebrahimnia, Sonia Alavi, Hamed Vaezi, Mahdieh Karamat Iradmousa, Azadeh Haeri

**Affiliations:** 1 Department of Pharmaceutics and Pharmaceutical Nanotechnology, School of Pharmacy, Shahid Beheshti University of Medical Sciences, Tehran, Iran; 2 College of Pharmacy, University of Illinois Chicago, Chicago, IL 60612, USA; 3 Protein Technology Research Center, Shahid Beheshti University of Medical Sciences, Tehran, Iran

**Keywords:** implantable drug delivery system, polymer depot, local drug delivery, controlled release, cancer chemotherapy

## Abstract

Conventional cancer chemotherapy regimens, albeit successful to some extent, suffer from some significant drawbacks, such as high-dose requirements, limited bioavailability, low therapeutic indices, emergence of multiple drug resistance, off-target distribution, and adverse effects. The main goal of developing implantable drug delivery systems (IDDS) is to address these challenges and maintain anti-cancer drugs directly at the intended sites of therapeutic action while minimizing inevitable side effects. IDDS possess numerous advantages over conventional drug delivery, including controlled drug release patterns, one-time drug administration, as well as loading and stabilizing poorly water-soluble chemotherapy drugs. Here, we summarized conventional and novel (three-dimensional (3D) printing and microfluidic) preparation techniques of different IDDS, including nanofibers, films, hydrogels, wafers, sponges, and osmotic pumps. These systems could be designed with high biocompatibility and biodegradability features using a wide variety of natural and synthetic polymers. We also reviewed the published data on these systems in cancer therapy with a particular focus on their release behavior. Various release profiles could be attained in IDDS, which enable predictable, adjustable, and sustained drug releases. Furthermore, multi-step or stimuli-responsive drug release could be obtained in these systems. The studies mentioned in this article have proven the effectiveness of IDDS for treating different cancer types with high prevalence, including breast cancer, and aggressive cancer types, such as glioblastoma and liver cancer. Additionally, the challenges in applying IDDS for efficacious cancer therapy and their potential future developments are also discussed. Considering the high potential of IDDS for further advancements, such as programmable release and degradation features, further clinical trials are needed to ensure their efficiency. The overall goal of this review is to expand our understanding of the behavior of commonly investigated IDDS and to identify the barriers that should be addressed in the pursuit of more efficient therapies for cancer.

See also the graphical abstract(Fig. 1).

## Introduction

As a leading cause of death, cancer presents a substantial barrier to extending life expectancy in every country across the globe. According to 2019 estimates by the World Health Organization (WHO), cancer is identified as either the first or second leading cause of premature death (occurring before the age of 70) in 112 of 183 countries worldwide and ranked either the third or fourth in an additional 23 countries (Sung et al., 2021[113]). The current diagnostic and therapeutic paradigms in the continuum of cancer care involve standardized screening measures for a few types of cancer, followed by a multimodal treatment approach composed of a combination of surgical resection, radiotherapy, and/or chemotherapy (Krukiewicz and Zak, 2016[60]; Magill et al., 2023[76]; Wolinsky et al., 2012[128]).

Even with the remarkable progress in early diagnosis and therapeutic modalities over recent years, which has substantially led to a steady decline in the incidence of cancer-associated mortality (Byers, 2010[18]), there are still undeniable shortcomings in rates of recurrence, treatment-related side effects, and morbidity with the present standard treatment regimen of most cancers. This is especially true for chemotherapy, which can be applied before or after surgical intervention, and with or without radiation therapy rather than surgery. Many current chemotherapeutic agents suffer from poor aqueous solubility which limits their intravenous (IV) delivery, unless they are modified chemically as a water-soluble pro-drug (as in the case of irinotecan) or formulated by using a surfactant/cosolvent-containing solution like ethanol/cremophor-EL (e.g. paclitaxel). Nevertheless, both approaches can result in poor bioavailability, hypersensitivity reactions, and other secondary adverse effects (Gelderblom et al., 2001[33]; Paulík et al., 2012[90]; Weiss et al., 1990[126]; Wolinsky et al., 2012[128]).

It is also imperative to mention that IV chemotherapy does not specifically target malignant cells, so it is very challenging to reach therapeutic levels of anti-cancer agents inside or adjacent to the tumoral area. Moreover, considerable concentrations of systemically administered chemotherapeutics frequently accumulate in normal tissues, leading to dose-limiting toxicity and serious side effects (Krukiewicz and Zak, 2016[60]). The use of systemic chemotherapy and radiation also substantially adds to the total treatment cost because of the huge material costs and the demand for strict cooperation among cancer specialists for drug delivery, management of side effects, and continuous monitoring. These therapeutic strategies are time-consuming for both patients and healthcare service providers, necessitating frequent visits over the treatment period (Wolinsky et al., 2012[128]).

**Figure 2:**
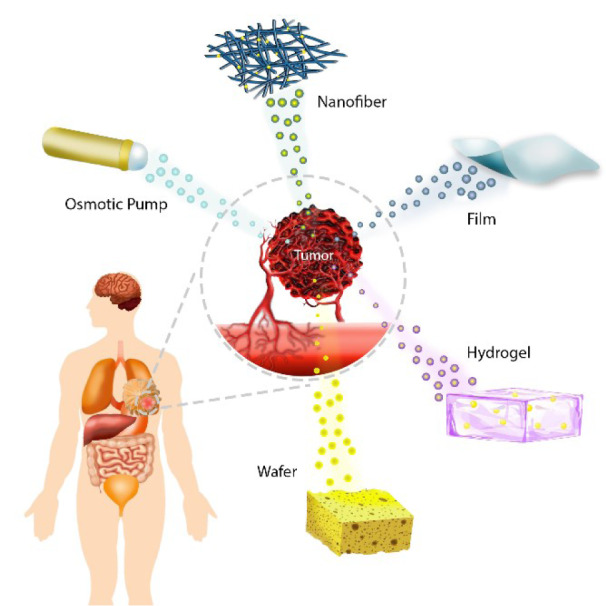
Application of different IDDS, including nanofibers, films, hydrogels, wafers, sponges, and osmotic pumps in tumor sites. The drugs integrated in these systems release locally into the target tumor site.

A rising approach to overcome some of the negative aspects of the present therapeutic paradigms involves the application of localized chemotherapy using implantable drug delivery systems (IDDS) aiming to increase the effectiveness of treatment and decrease patient morbidity. IDDS are administered directly at the tumoral site, possessing several distinct advantages over conventional systemic delivery (Magill et al., 2023[76]), such as i) loading of poorly water-soluble antineoplastic drugs (Monterrubio et al., 2016[81]); ii) stabilization of embedded chemotherapeutic molecules and maintenance of their anti-cancer efficacy (Wolinsky et al., 2010[129]); iii) prolonged and controlled drug release to yield adequate tumoral uptake (Sun et al., 2013[111]); iv) possibility of one-time administration of drugs (Magill et al., 2023[76]); v) direct delivery to the tumoral sites (Wu et al., 2018[133]), and vi) reduced adverse effects because of the avoidance of systemic blood circulation of anti-cancer drugs (Zhang et al., 2017[149]). Numerous types of synthetic and natural-based polymers have been assessed as controlled-release depot systems for cancer drug delivery, such as chitosan (Puente et al., 2018[97]), gelatin (Jaiswal et al., 2013[50]), hyaluronic acid (HA) (Fong et al., 2017[31]), silk (Yavuz et al., 2018[139]), poly(ε-caprolactone) (PCL) (Babadi et al., 2022[12]), poly(lactic acid) (PLA) (Monterrubio et al., 2016[81]), poly(D,L-lactide-co-glycolide) (PLGA) (Lei et al., 2013[64]), and poly(vinyl alcohol) (PVA) (Zhang et al., 2017[149]). Here, we discuss the current research and clinical landscape of IDDS, including nanofibers, films, hydrogels, wafers, sponges, and osmotic pumps for local delivery of chemotherapeutics (Figure 2(Fig. 2)). We summarize our analysis in relevant tables to delineate key clinical areas that IDDS have found remarkable success and further highlight challenges that may impede their future clinical translation.

## Nanofibers

Drug-loaded electrospun nanofibers are one of the most effective and extensively studied IDDS due to their distinctive properties, such as high porosity and surface area, programmable and predictable drug release profile, efficient encapsulation of the cargoes, biocompatibility, and high drug loading (Abid et al., 2019[2], Khodadadi et al., 2020[57]). By using these nanocarriers, high local concentration of the chemotherapeutic drug is achieved within the tumor region, while systemic exposure is minimized (Zhang et al., 2016[142]), which can lead to better therapeutic responses and lower incidence of adverse effects (Guimarães et al., 2015[36]; Luo et al., 2012[74]). In addition, the complex and interconnected structure allows electrospun nanofibers to provide an additional advantage in local cancer therapy, imitating the topography of the extracellular matrix (ECM) (Iqbal et al., 2017[48], Khodadadi et al., 2020[57]). It is pertinent to mention that ECM degradation is one of the crucial reasons for cancer metastasis due to its vital role in controlling cell proliferation and differentiation (Elgundi et al., 2019[29]). Therefore, local implantation of the electrospun nanofibers can overcome the absence of ECM and diminish tumor recurrence/metastasis risk (Sun et al., 2019[112]). 

Several methods are utilized for nanofiber preparation, including electrospinning, melt-blowing, self-assembly, and forcespinning. Electrospinning exhibits many advantages, such as the ability to produce a wide range of fiber sizes, scale-up feasibility, low setup cost for laboratory scale experiments, and versatile fiber compositions. Therefore, electrospun nanofibers are extensively used in different fields, including wound healing, cancer treatment, tissue engineering, and regenerative medicine (Chen et al., 2020[23]).

Many polymers have been exploited to create nanofibers for anti-cancer drug delivery (Table 1(Tab. 1); References in Table 1: Babadi et al., 2022[12]; Cen et al., 2020[19]; Chen et al., 2021[22]; Graham-Gurysh et al., 2018[35]; Hobzova et al., 2019[44]; Hsu et al., 2021[46]; Jain et al., 2014[49]; Kaplan et al., 2016[52]; Li et al., 2022[67]; Liu et al., 2021[69]; Luo et al., 2012[73][74]; Ma et al., 2015[75]; Mazza et al., 2019[78]; Monterrubio et al., 2016[81]; Samadzadeh et al., 2021[102]; Sun et al., 2019[112]; Wang et al., 2020[123]; Xu et al., 2022[135]; Yang et al., 2015[136]; Zhang et al., 2015[148], 2016[142][147], 2017[149]), among which the typical ones are PCL (Talimi et al., 2023[115]), PLA (Monterrubio et al., 2016[81]), PLGA (Tseng et al., 2015[120]), and PVA (Zhang et al., 2017[149]).

PCL, an aliphatic polyester, is one of the most widely applied polymers for various medical purposes because of its biocompatibility, biodegradability, safe degradation products, and approval by Food and Drug Administration (FDA) (Abid et al., 2019[2]). In comparison with PLA nanofibers, PCL ones have been reported to be more hydrophobic in nature, which makes them appropriate for more prolonged sustained release than their PLA-based counterparts. Thus, PCL nanofibers can be utilized for malignant cells requiring longer periods of treatment (Abid et al., 2019[2]). In a recent study, a series of epirubicin-loaded PCL/PLGA nanofibers having tunable rates of drug release and degradation was developed by emulsion electrospinning technology and characterized for local chemotherapy (Sun et al., 2019[112]). These vehicles had distinctive core-sheath structures consisting of 0, 5, or 10 wt% of epirubicin in the core and 15 or 25 wt% of PCL in the sheath to optimize the antitumor effect. The findings of this study indicated that the drug release and degradation of the proposed carriers could be reduced by increasing the content of PCL in the sheath, leading to improved anti-cancer activity (Sun et al., 2019[112]). 

**Figure 3:**
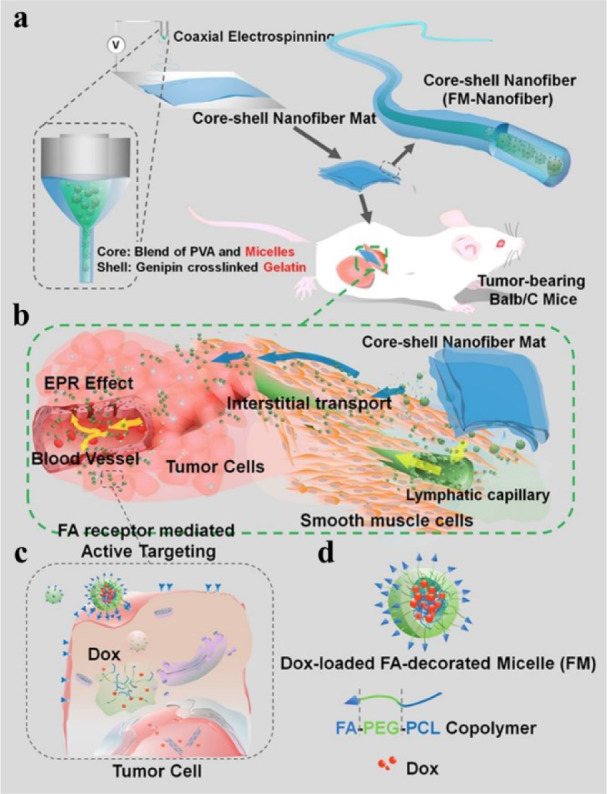
Schematic illustrations of preparation and delivery of core-shell nanofibers to cancer cells. (a) Core-shell nanofiber mats are fabricated using coaxial electrospinning. Doxorubicin-loaded micelles are incorporated in the core of the coaxial electrospun nanofibers. The nanofiber shells are made of genipin-crosslinked gelatin. (b) After implantation in tumor-bearing mice, the micelles are released from the nanofiber and reach the tumor site by mechanisms such as enhanced permeation and retention (EPR) and interstitial transport. (c) The micelles bind to folate receptors on the tumor cells using their folic acid (FA) ligands, and transport doxorubicin into the cancer cells. (d) Schematic structure of the micelles. Reprinted from Yang et al. (2015). Copyright (2015) with permission from American Chemical Society.

To achieve a more sustainable drug release rate, drugs could be encapsulated inside vesicular systems such as micelles or liposomes, which are later loaded inside the nanofibers (Poláková et al., 2019[94]). As an example of this approach, an active-targeting micelles-in-nanofiber implantable device was fabricated to treat cancer safely and effectively. The hydrophobic doxorubicin was incorporated inside micelles, which were self-assembled using amphiphilic folate-conjugated PCL- poly(ethylene glycol) (PEG) copolymers. Later, the core-shell nanofibers were made up via coaxial electrospinning, using the blend of micelles and PVA aqueous solution as the core and genipin-crosslinked gelatin as the shell of the nanofibers. Functionalizing the micelles with folate increased their targeting effects. The release of doxorubicin inside the micelle-loaded nanofibers reached about 40 % in 2 days and approximately 80 % in 12 days. Loading doxorubicin-encapsulated micelles inside the core-shell nanofibers prolonged the release time and decreased the initial burst release of doxorubicin compared to free micelles (Figure 3(Fig. 3)) (Yang et al., 2015[136]).

As another FDA-approved biopolymer, PLA, consisting of lactic acid monomers, has been shown to be non-toxic and to possess good compatibility with drugs (Abid et al., 2019[2]). Based on the chiral feature of the structural unit, this polymer can be divided into three stereoisomers, i.e., poly(L-lactic acid) (PLLA), poly(D-lactic acid) (PDLA), and poly(D,L-lactic acid) (PDLLA). PLLA and PDLA are semicrystalline and crystalline materials, respectively, and both have regular chain structures, whereas mixing the D and L isomers results in an amorphous polymer. Since PLLA degrades to L(+) lactic acid, which is the naturally occurring form of lactic acid, and shows higher mechanical strength than PDLA, it is preferred over PDLA. PDLLA is amorphous and could be useful for drug-eluting applications (Hadasha and Bezuidenhout, 2018[38]). The crystalline form of PLA exhibits higher chemical stability, and lower water resistance and biodegradation speed than the amorphous form (Kühnert et al., 2018[61]).

Studies using PLA polymer have met with different degrees of success. For instance, Monterrubio and colleagues addressed the incorporation of SN-38 microcrystals into PLA nanofibers to improve both its poor aqueous solubility and toxicity profile for the efficient control of pediatric solid tumors following subtotal resection surgery. Phosphate buffer solution (PBS) at pH 7.4 with or without 2-hydroxypropyl-β-cyclodextrin (HPCD) as a solubilizer was used as the release medium. Obtained results showed that in the presence of HPCD, the PLA matrices released their content completely within 24 h. Moreover, *in vivo* experiments showed a significant reduction in tumor size and delayed tumor growth in mouse models after subtotal bilateral tumor resection (Monterrubio et al., 2016[81]). 

In another study, the effect of the number of layers on the release behavior of nanofiber mats was assessed by Zhang and co-workers (2016[147]). They prepared dual drug-encapsulated nanofiber mats with four layers. Oxaliplatin and dichloroacetate (DCA) were sequentially electrospun into the discrete layer of developed fabrics made from PLLA and the oxaliplatin-incorporated fibers' layer was situated between two nanofibrous layers and the basement layer (PLLA film). *In vitro* release studies demonstrated that in comparison with DCA-loaded monolayered PLLA fibers (~ 75 % in 24 h) and oxaliplatin-loaded monolayered PLLA fibers (~ 40 % in 24 h), the co-loaded multilayered mats exhibited a much slower release trend for both drugs, especially oxaliplatin (~ 70 % and ~ 1 % in 24 h, for DCA and oxaliplatin, respectively) (Zhang et al., 2016[147]).

PLLA nanofibers were also employed as carriers to co-deliver doxorubicin and multi-walled carbon nanotubes (MWCNTs) for combined chemo- and photothermal cancer therapy (Zhang et al., 2015[148]). Carbon nanotubes are efficient thermal generators via absorbing near-infrared radiation (NIR). Based on the findings of this study, it was revealed that NIR could not only trigger burst release of doxorubicin molecules from the nanofibers because of the rather low glass transition temperature of PLLA, but also considerably raise the temperature of fibers-covering tumor area, leading to promising *in vitro* and *in vivo* results (Zhang et al., 2015[148]). However, possible carbon nanotube-induced toxicity is an important issue. Many factors could increase the toxicity of carbon nanotubes, such as high length, presence of metal impurities, and increased aggregation state (Alshehri et al., 2016[6]). High length and metal impurities could also decrease the biodegradability of carbon nanotubes, which increases their lifetime in living systems, contributing to potential long-term toxicity (Yang and Zhang, 2019[137]). The high aggregation degrees of MWCNTs could also increase their accumulation in organs, such as livers and lungs, and cause inflammatory responses (Alshehri et al., 2016[6]). In the mentioned article, it was observed that the adverse effects of the fabricated system on livers and kidneys were insignificant due to prolonged degradation of PLLA and therefore, concentration of MWCNTs was low enough with appropriate blood clearance and reduced aggregation (Zhang et al., 2015[148]).

In a recent study, PDLLA/PEG micro/nanofibers loaded with paclitaxel were developed by needleless electrospinning technology, which provides large-scale production (Hobzova et al., 2019[44]). In this study, the effect of the addition of PEG of different molecular weights (6, 20, and 35 kDa) to modify the release pattern of a hydrophobic drug (paclitaxel) was investigated. According to the results, it was found that low amounts of drug released from the pure PDLLA fibers, and the addition of PEGs considerably increased the released amounts of drug and also extended its release period. This influence was more noticeable by PEG of the lowest molecular weight in the early phase of release profiles (Hobzova et al., 2019[44]). Even though PEG is biocompatible, it is not degradable *in vivo* and is mostly eliminated from the body via the kidneys. To increase the degradability of the systems fabricated with PEG, some studies have prepared co-polymers consisting of PEG and a biodegradable polymer, such as PLA (Peng et al., 2016[91]).

PLGA, the copolymer of PLA and poly(glycolic acid), has also been explored by numerous research groups to develop nanofibers for a wide range of therapeutic applications. This is because this FDA-approved polymer is also biocompatible and biodegradable, and its degraded products (i.e., lactic acid and glycolic acid) are finally converted into carbon dioxide and water and eventually eliminated (Chereddy et al., 2016[25]). In a recent study, PLGA nanofibrous films were developed to prevent postoperative cancer recurrence and metastasis. Doxorubicin-loaded microparticles were co-delivered with aspirin for eliminating cancer cells and inhibiting platelet-triggered proliferation, simultaneously. The PLGA nanofibrous films enhanced the accumulation of the drugs inside tumor resection cavities and enabled a sustained release manner for both doxorubicin microparticles and aspirin. While the release percentage of doxorubicin microparticles reached about 59 % in 24 hours, the same approximate amount of aspirin was released in 120 hours. This difference in release rate contributed to the deactivation of platelets over an extended period while maintaining efficient tumor-killing properties (Li et al., 2022[67]).

A study explored the idea of using PLGA nanofibers for extended delivery of carmustine, irinotecan, and cisplatin in the cerebral cavity, aiming at successful treatment of glioblastoma multiforme (GBM). The nanofibrous membranes prepared in this work showed the early release phase of irinotecan within 7 days and carmustine and cisplatin within 4 days. This was most likely related to the relatively low aqueous solubility of the loaded pharmaceuticals. However, it is important to note that the *in vivo* experiments showed no obvious initial burst release. This was probably attributable to the difficult penetration of the released drug molecules across the blood-brain barrier, leading to their accumulation in the brain for a prolonged period. It was also revealed that carmustine and irinotecan were gradually released over the first two weeks, and thereafter their release rate increased and remained high for four weeks. In contrast, cisplatin was released at a high rate and remained at a plateau of high concentration within the first four weeks, and after that, its release rate steadily slowed down. Therefore, combinatorial delivery of cisplatin, carmustine, and irinotecan using PLGA nanofibrous membranes demonstrated a complementary effect for the efficient treatment of GBM (Tseng et al., 2015[120]).

PVA, another commonly used biocompatible polymer, has also been extensively studied for the fabrication of nanofibers due to its high hydrophilicity, excellent fiber-forming ability, and biocompatibility (Nitanan et al., 2013[84], Steffens et al., 2020[109]). An interesting study developed an implantable Pt(IV) micelle/DCA co-encapsulated nanofiber membrane for enhanced local chemotherapy. For this purpose, reduction-responsive micelles were synthesized by polymerization of PEG_2k_ units and Pt(IV) prodrug, and co-electrospun with DCA into PVA nanofibers. The release behavior of cargoes from the fibers was first studied without a dialysis bag in PBS (pH 7.4), and it was found that nearly 70 % of Pt and 90 % of DCA were released during the first hour. Because of the uniform distribution of micelles and DCA in the matrix, the fast-release patterns were explained by the fast dissolving of PVA. The release pattern of Pt from the nanofibers was investigated by the dialysis bag technique to guarantee the collection of small molecule Pt in the release medium. The release rate of Pt was found to be very slow, so only 20 % was released in PBS (pH 7.4) after 72 h. In this study, to further evaluate the reduction-sensitive release of Pt and simulate the condition in tumor cells, sodium ascorbate (NaVc) and acetate buffer solution (pH 5.0) were applied. The results showed that 40 % and 57 % of Pt were released in acetate buffer solution without and with NaVc, respectively, displaying the high reduction-responsiveness of the prodrug-backboned micelles triggered by NaVc (Zhang et al., 2017[149]).

The nanofiber-based IDDS studied in this part illustrate that the physicochemical diversity of anti-cancer drugs with respect to important parameters like aqueous solubility, size, degree of ionization, environmental conditions, and process parameters can greatly influence their release profiles (Adepu and Ramakrishna, 2021[3]). The drug release profile of nanofibers consists of three main stages. The first stage is when the release happens from the nanofiber surface. In the second stage, the drug which is loaded inside the polymer matrix diffuses to the surface of the nanofibers and is subsequently released. The third stage of drug release happens as a result of the degradation or decomposition of the nanofibers (Poláková et al., 2019[94]). To develop nanofibers with immediate drug release, highly porous polymers with interconnected pores and large specific surface areas should be employed (Adepu and Ramakrishna, 2021[3]; Sharifi et al., 2022[103]). Many strategies could be used to modify the drug release from nanofibers. For example, fabricating sandwich nanofibrous structures where the drug-incorporated layer overlaps with the other layers and the drug release is prolonged due to slow liquid convection (Poláková et al., 2019[94]). Furthermore, in core-shell nanofibers, sustained drug release is obtained when the drugs in the core phase with the highest concentration diffuse out of the polymer matrix (Monfared et al., 2019[80]). Other techniques for controlling the drug release from nanofibers include adjusting the composition and drug-to-polymer ratio, choice of polymer and excipients, structure, diameters, swelling, and thickness of the nanofibers (Rasouli et al., 2019[99]). For instance, water-soluble polymers exhibit immediate release while degradable or swellable polymers show prolonged drug release (Singh et al., 2021[104]). In addition, drug release could be controlled by preparing stimuli-responsive nanofibers. These types of nanofibers could release the drug in response to stimuli such as pH, temperature, light, electric field, magnetic field, or a combination of different stimuli (Weng and Xie, 2015[127]).

## Films

Polymeric films have also been explored extensively as local implantable scaffolds to deliver a variety of anti-cancer agents and have displayed promising results *in vitro*, as well as *in vivo* (Table 2(Tab. 2); References in Table 2: Liu et al., 2010[72], 2012[71]; Sonvico et al., 2018[107][108]; Tian et al., 2021[118]; Wolinsky et al., 2010[129]; Wu et al., 2018[133]; Zhang et al., 2016[143]). Implantable films possess some benefits for tumor drug delivery, such as achieving concentrated and constant drug delivery to malignant tissues, minimizing systemic side effects, and improving tumor cytotoxicity by prolonging the release of chemotherapeutics locally and ensuring that residual tumor cells are exposed to them during multiple cell cycles (Karki et al., 2016[55]; Liu et al., 2012[71], 2010[72]; Sonvico et al., 2018[108]; Wolinsky et al., 2012[128]). Furthermore, the versatility of design of film implants and the elasticity of polymeric materials employed for their development allows them for sufficient coverage of surface, fixation, and diffusion of drug molecules within the sites at greatest risk for local tumor recurrence (Wolinsky et al., 2012[128]). Considering such advantageous properties, polymeric films have been well examined against a variety of malignancies like lung cancer (Wolinsky et al., 2010[129]), prostate cancer (Wu et al., 2018[131]), melanoma (Zhang et al., 2016[143]), sarcoma (Liu et al., 2012[71]), and malignant pleural mesothelioma (Sonvico et al., 2018[107]). 

**Figure 4:**
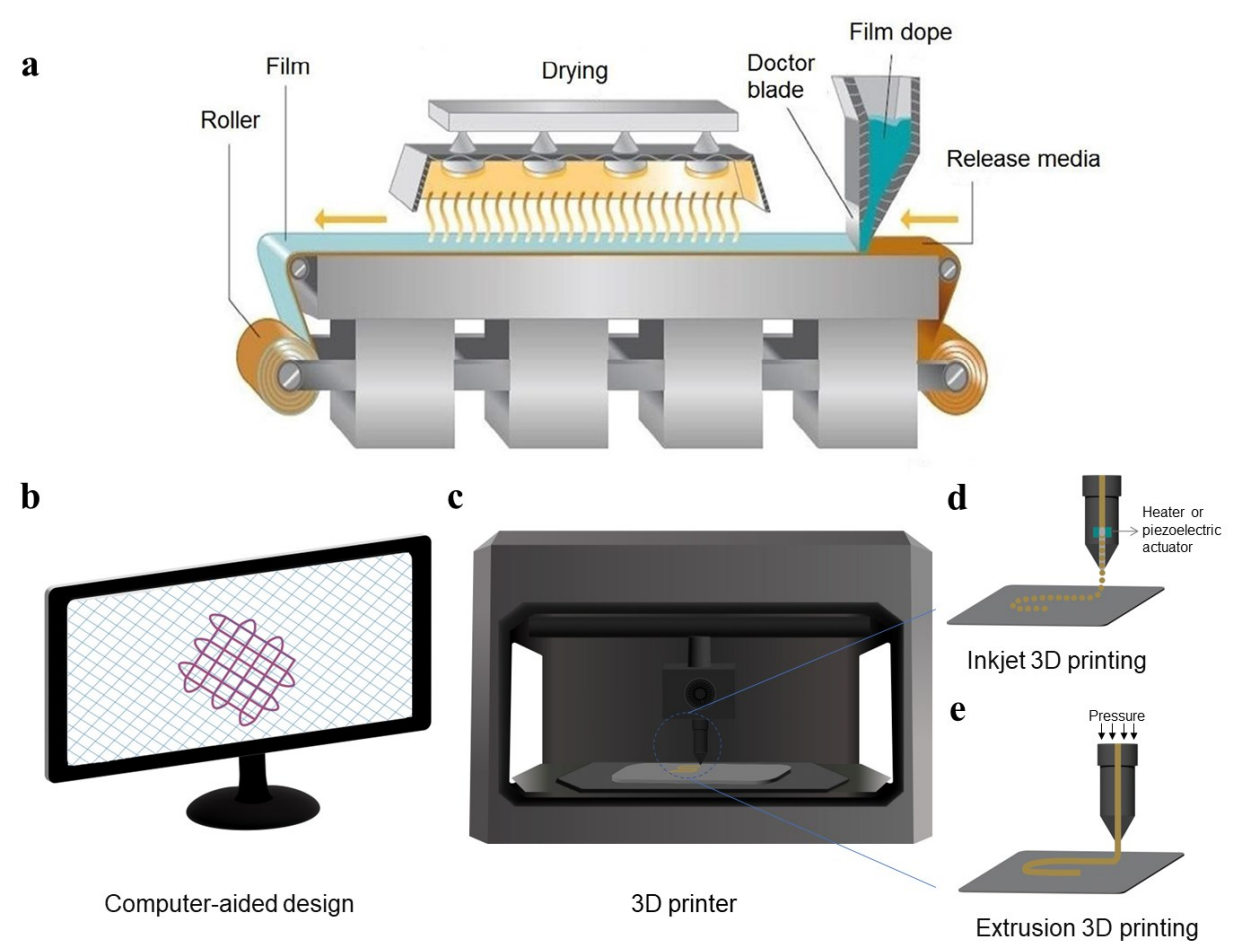
Film preparation methods. (a) Illustration of solvent-casting-based film manufacturing. Reprinted with permission from (Amin et al., 2015). (b to e) Film preparation by 3D printing. (b) The computer-aided design of the desired shape of the system. (c) The 3D printer. (d) Inkjet 3D printing which consists of a heater or a piezoelectric actuator to eject the ink drop-wise. (e) Extrusion 3D printing which deposits the ink continuously.

Films could be prepared using several fabrication methods. Solvent-casting is one of the most preferred methods of film manufacturing due to its feasibility and low-cost process (Figure 4a(Fig. 4); Reference in Figure 4: Amin et al., 2015[8]). In this method, the polymeric solution is cast into a substrate and the solvent is then evaporated by drying, which leaves a drug-loaded polymeric film (Babadi et al., 2022[13], Hosseinpour-Moghadam et al., 2021[45], Sonvico et al., 2018[107]). Hot melt extrusion is another common film preparation method which in contrast to solvent-casting, does not require organic solvents (Karki et al., 2016[55]).

Printing technologies, including flexographic, inkjet, and other 3D printing methods are relatively novel techniques for preparing polymeric films, which have gained interest due to their cost-effectiveness and flexibility (Figure 4b-e(Fig. 4)). Flexographic printing uses contact printing to transfer active ingredients into films which is suitable for heat-sensitive products. However, this method suffers from certain disadvantages, such as low resolution and high risk of contamination. Inkjet printing is an accurate and versatile method that is highly applicable for manufacturing low-dose or personalized medicines (Karki et al., 2016[55]). 3D printing constructs 3D objects with diverse geometries and materials using computer-aided design models (Figure 4b(Fig. 4)). 3D printing can also be utilized in combination with conventional methods to produce films (Preis et al., 2015[96]). For instance, in a recent study, mucoadhesive local vaginal films containing disulfiram were prepared for treating cervical cancer using 3D printing and hot-melt extrusion (Almotairy et al., 2023[5]).

Generally, there are two main categories of polymeric materials utilized in the development of film implants for cancer therapy: non-biodegradables (e.g., polyurethane (PU)) and biodegradables (e.g., PLA, PCL, and CS) (Table 2(Tab. 2)). As an example of implants fabricated with non-biodegradable polymers, Zhang and co-workers studied the release of bioactive peptides from thermoplastic PUs (TPUs) with various hard and soft segments: Tecoflex 80A (T80A) and Elast-Eon 5-325 (E5-325) (Zhang et al., 2016[143]). TPUs show high biocompatibility and suitable mechanical properties, which makes them favorable polymers for fabricating IDDS. They are composed of soft and hard thermodynamically incompatible segments, which form micro-domains by undergoing phase separation. At room temperature, the highly polar hard segments induce non-covalent crosslinking between polymer chains, whereas the soft segments provide flexibility. Micro-domains could be formulated to interact with different drug molecules that suit their physicochemical properties. According to the findings of Zhang et al., peptide release was found to be dependent on both the size and the TPU composition. T80A exhibited a more rapid release profile than E5-325, which was associated with the degree of hydration. Moreover, the medium composition affected both the extent and rate of peptide efflux. It was also indicated that enhanced control of peptide efflux, particularly the early burst effect was achieved by blending the different TPUs. The group also assessed the influence of TPU-impregnated PMX53, an anti-inflammatory cyclic peptide, on the B16-F10 melanoma cancer model in C57BL/6 mice. Based on their results, elevated PMX53 plasma levels were maintained for at least nine days when using a mixture of T80A and E5-325A (50:50), and a notable reduction in cancer cell proliferation was also observed (Zhang et al., 2016[143]). 

When designing IDDS with non-biodegradable polymers, it is important to consider that these systems require removal strategies. Though non-biodegradable films can work as efficient reservoirs, their surgical requirements make these systems invasive and uncomfortable for patients. Thus, it would be expected that the body responds more efficiently when treated by biodegradable films, thanks to their inherent nature and much fewer surgical concerns (Magill et al., 2023[76]; Stewart et al., 2020[110]). Liu et al. fabricated paclitaxel poly(glycerol monostearate co-ɛ-caprolactone) (PGC-C18) films and employed this platform to prevent postoperative recurrence in non-small-cell lung cancer. The modification by stearic acid provided a controlled release of paclitaxel (cumulative ~ 31 % drug release at day 50), thus prolonging the pharmaceutical effect. Ten days after treatment, a 3000-fold higher drug concentration at the site of tumor resection was achieved with film implantation compared to systemic administration. In addition, 22 % and 83 % of mice receiving systemic therapy and film implantation were free from recurrence following surgery, respectively (Liu et al., 2010[72]). Consequently, this research group also applied this platform in the recurrent sarcoma model, and a significant improvement in survival rate and a decrease in locoregional recurrence were observed (Liu et al., 2012[71]).

In another study regarding biodegradable polymers, Tian et al. prepared trilayered films to be utilized as biliary stents for treating cholangiocarcinoma and inhibiting biofilm formation. The films contained two outer ofloxacin-loaded and paclitaxel-loaded PLA layers, isolated by another middle PLA layer which enabled a unidirectional release from each drug-loaded layer in opposite directions. All the films with different compositions showed an initial rapid drug release followed by a slower release in later stages. Furthermore, incorporating different amounts of the drugs or PEG in the films could affect the paclitaxel release pattern. For example, films without PEG content exhibited a cumulative paclitaxel release of 3.61 % after 106 days, while adding 20 % of PEG with an average number molecular weight (M_n_) of 1500 increased the cumulative release to 18.33 %. Also, increasing the loaded drug in the films containing paclitaxel or ofloxacin alone increased the cumulative release of the drugs. The *in vitro* results indicated that the films successfully inhibited tumor cell proliferation and biofilm formation (Tian et al., 2021[118]). Furthermore, PEGs with M_n_ of 1500 Da are cleared through kidneys (Hoang Thi et al., 2020[43]), no concerns remain about the excretion of the IDDS from the body in Tian's study.

Considering the film implants reviewed in this section, the release behavior of these systems differs markedly, depending on the type of polymers used for their development and the cargoes. For example, the release of pemetrexed from the hyaluronate-based film was found to be almost complete within 2 h, which lacked control on release due to high solubility and low molecular weight of pemetrexed and its lack of electrostatic attractions with the polymers in the film (Sonvico et al., 2018[107]), while paclitaxel-loaded PLA-PEG (20 %) film released only about 30 % of paclitaxel within 300 days, without reaching a plateau (Wu et al., 2018[131]). Using a PLA-based drug-free backing layer enabled a unidirectional drug release from the side in contact with tumor cells, which increased drug accumulation in the cancer site (Wu et al., 2018[131]). However, it could be advisable to monitor the degradation rate of the system from the body by *in vivo* imaging and discuss it with regard to the drug release profile. Furthermore, *in vitro* release tests could not simulate the degradation of biodegradable systems; therefore, the actual *in vivo* release profiles could be different from observed *in vitro* data.

The release behavior is also highly related to the structure of polymers. For instance, the dissolving speed of linear amorphous polymers is much higher than semi-crystalline or crosslinked polymers. The drug release is remarkably affected by the erosion of the films. Furthermore, the quantity of plasticizer used in the formulations could slightly enlarge the film thickness, which correlates with the drug amount incorporated inside the film. Film thickness needs to be suitable for facile administration (Karki et al., 2016[55]). In the articles discussed in this study, films with different thicknesses such as 40 µm (Liu et al., 2010[72]; Wolinsky et al., 2010[129]), 200 µm (Park et al., 2015[89]), and 1-2 mm (Zhang et al., 2016[143]) were fabricated. There are a few studies regarding the effects of film thickness on the disintegration and dissolution times of films (Zhang et al., 2018[144]). Therefore, more studies on the optimization of film thickness for IDDS should be performed to adjust the release profile and minimize tissue injury. 

Apart from endowing unidirectional release, fabricating multi-layered films with different drug-incorporated layers enables drug release with different rates from each layer, which could result in multi-stage release profiles (Rong et al., 2012[100]). Also, hybrid systems of nanoparticle-embedded films can be designed to further extend the release duration (Pereira et al., 2016[93]) or achieve multi-step release profiles.

## Hydrogels

**Figure 5:**
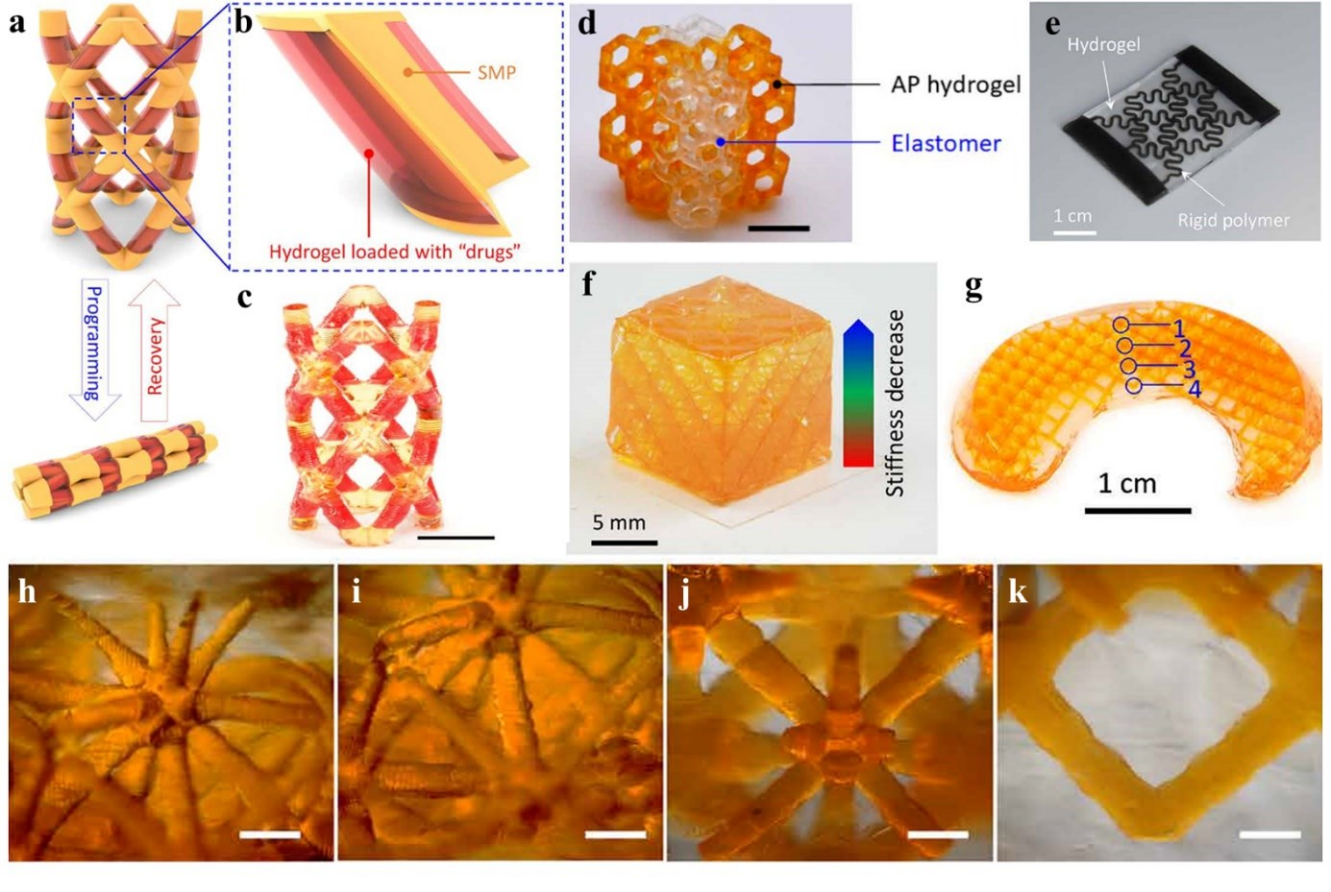
3D printed hydrogel-based structures. (a-c) A printed cardiovascular shape memory polymer (SMP)-hydrogel stent. (a) Design of the stent with the ability to turn into a squeezed shape and recover into its original shape based on temperature. (b) A detailed illustration of the design showing the drug-incorporated hydrogels are integrated into SMP rods. (c) The printed SMP-hydrogel stent. (d) A diagonally symmetric Kelvin structure consisting of elastomer and acrylamide-poly(ethylene glycol) diacrylate (PEGDA)(AP) hydrogel. (e) A hydrogel composite strengthened by a rigid horseshoe polymer structure. (f) A printed hydrogel cube possesses gradient stiffness and is strengthened by a rigid lattice polymer structure. (g) A printed hydrogel meniscus strengthened by a rigid lattice structure. (h-k) The microscopic images of the meniscus at locations number 1 to 4, respectively. Reprinted with permission from (Ge et al., 2021).

Hydrogels are semi-solid structures consisting of hydrophilic polymers, which can absorb large amounts of water, maintaining a 3D network because of the development of internal crosslinking bonds (Ho et al., 2022[42]). Physical and chemical crosslinking are among the common methods for preparing hydrogels. Physically crosslinked hydrogels are relatively facile to produce and are generally a result of ionic interactions, heat-induced aggregation, hydrogen bonding, and heating or cooling polymer solutions. Chemical crosslinking involves the reaction of the functional groups on polymer backbones to link polymer chains together. Crosslinkers, including glutaraldehyde (GA) and epichlorohydrin are utilized for obtaining chemically crosslinked hydrogels of various polymers (Gulrez et al., 2011[37]). For instance, in a study by Puente et al., chitosan hydrogels were crosslinked by GA (Puente et al., 2018[97]). However, GA is a toxic substance that could inhibit cell growth even when used at low concentrations. In a study conducted by Yu et al., crosslinking of gelatin hydrogels with a low concentration of GA induced a significant foreign body response and inflammation upon subcutaneous implantation in mice (Yu et al., 2016[140]). Since other crosslinking agents are also mostly toxic and could change the integrity of materials, the removal of any remaining unreacted agents from the chemically crosslinked hydrogels is very important. Using alternative methods that avoid the use of crosslinkers (e.g., physical crosslinking) has increasingly gained interest over the years (Hennink and van Nostrum, 2002[40]). Grafting, radiation crosslinking, interpenetrating polymer networks, and hydrophobic interactions are other methods to produce hydrogels (Gulrez et al., 2011[37]). Noncovalent molecular self-assembly assisted by shape complementarity or nucleic acids is another hydrogel preparation strategy that is mostly applied for macromolecule-based hydrogels (Zhang and Khademhosseini, 2017[145]). Song et al. prepared a self-assembled hydrogel loaded with succinated paclitaxel which could inhibit cancer cell proliferation *in vitro* (Song et al., 2018[106]). 3D printing is another easy technique to generate hydrogels with complex structures with high precision and flexibility (Figure 5(Fig. 5); Reference in Figure 5: Ge et al., 2021[32]). In a recent study, gemcitabine-loaded coaxial hydrogel patches were prepared using 3D printing which effectively inhibited pancreatic tumor cell growth (Talebian et al., 2021[114]). Furthermore, microfluidic printheads can deliver dual material types to produce multimaterial hydrogels with higher printing speed and enhanced shifting between the materials (Zhang and Khademhosseini, 2017[145]). 

Hydrogels have been of significant interest for a diverse range of biomedical and pharmaceutical applications thanks to their biodegradability, excellent flexibility, shape-adaptive function, and minimum invasive administration (Gulrez et al., 2011[37]; Li and Mooney, 2016[68]). Recently, there have been notable advances in utilizing hydrogels for the management of a variety of cancers (Table 3(Tab. 3); References in Table 3: Cheng et al., 2013[24]; Fong et al., 2017[31]; Hu et al., 2021[47]; Jaiswal et al., 2013[50]; Jing et al., 2021[51]; Lee et al., 2019[63]; Li et al., 2020[66], 2023[65]; Liu et al., 2019[70]; Nieto et al., 2022[83]; Peng et al., 2014[92]; Puente et al., 2018[97]; Song et al., 2021[105]; Talebian et al., 2021[114]; Wang et al., 2022[125]; Wu et al., 2018[133], 2019[132]; Xu et al., 2021[134]; Zhuang et al., 2020[152]), such as breast cancer (Fong et al., 2017[31]; Jaiswal et al., 2013[50]; Liu et al., 2019[70]; Zhuang et al., 2020[152]), lung cancer (Lee et al., 2019[63]), hepatocellular carcinoma (Peng et al., 2014[92]), GBM (Puente et al., 2018[97]), and osteosarcoma (Wu et al., 2018[133]). Based on the polymer origin, these hydrogels can be categorized into three major groups: synthetic, natural, and natural/synthetic hybrid hydrogels (Bashir et al., 2020[14]).

Hydrogels from natural sources are usually derived from proteins and polysaccharides (Bashir et al., 2020[14], Ho et al., 2022[42]). Among various proteins capable of forming into hydrogels for cancer therapy, gelatin, is one of the most popular ones, which has been widely investigated either alone or in combination with other polymers by numerous research groups (Jaiswal et al., 2013[50]; Lee et al., 2019[63]; Wu et al., 2018[133]). One of these studies addressed the incorporation of doxorubicin into a semi-interpenetrating hydrogel network of gelatin and poly(acrylic acid) (PAA) to engineer an efficient system, which can be administered as a post-surgical implant for solid tumors (Jaiswal et al., 2013[50]). In this study, gelatin was kept free, whereas PCL diacrylate was used as a crosslinking agent for PAA chains with concentrations of 0.2 - 2 mol %, which can offer control over the drug release as well as the degradation behavior of the device. Therefore, the impact of crosslinker concentration on the key physicochemical characteristics of the system was also investigated. From the *in vitro* release experiments in PBS (pH 6.5), it was found that although there was a controlled release pattern for all of the matrices over 30 days, by increasing the concentration of crosslinker from 0.2 to 2 mol %, the release rate decreased from ~ 70 % to ~ 30 % (Jaiswal et al., 2013[50]). This can be justified by a higher crosslinking degree making the network denser and resulting in a slower release of drug molecules (Khan and Ranjha, 2014[56]). Furthermore, buffer media penetration inside the polymeric network was suggested to be the rate-determining step for the release of doxorubicin, and doxorubicin was released afterward by overcoming its interactions with the functional groups of the polymers. Also, the matrices using the lowest crosslinker concentration (0.2 mol %) degraded completely in 20 days *in vitro*, while matrices prepared with higher concentrations of the crosslinker only degraded from 12 % to 28 % within 30 days (Jaiswal et al., 2013[50]).

A variety of polysaccharides like chitosan (Puente et al., 2018[97]), gellan gum (Nieto et al., 2022[83]), HA (Fong et al., 2017[31]), and alginate (Brudno et al., 2018[17]) have also been explored as hydrogels for cancer drug delivery. These hydrogels can be fabricated by methods including covalent crosslinking, polymerization, and esterification (Ho et al., 2022[42]). A study reported the use of an injectable chitosan hydrogel implant that released a chemotherapeutic agent (temozolomide) while maintaining radioactive agents (iodine-131, ^131^I) in the cancer site to enhance the local control and treatment results of GBM (Puente et al., 2018[97]). According to the findings of this study, temozolomide was fully released over the first 2 days with a negligible release of ^131^I within 42 days. Moreover, *in vivo* experiments showed that ^131^I was totally retained in the cancer site with very limited distribution in normal tissues. This was achieved by conjugating ^131^I to human serum albumin (HSA), which is a large and biocompatible molecule, and subsequently encapsulating the iodinated HSA inside biodegradable, crosslinked alginate microparticles to further immobilize the HSA-conjugated ^131^I molecules and prevent their release. Moreover, ^131^I exhibits a relatively short half-life of 8 days, which allows it to decay while still encapsulated inside the hydrogel. Furthermore, when administered locally, temozolomide accumulated in the cancer site at 10-fold greater concentrations compared to when administered systemically (Puente et al., 2018[97]). In another study, redox-responsive gellan gum-based hydrogels incorporated with paclitaxel were developed for treating HER2-positive breast cancer. The hydrogels were crosslinked with different degrees using 1.5, 3, or 4.5 mg/ml of L-cysteine to enhance their stability and make the hydrogels redox-responsive. The hydrogels were prepared in PBS or acetate buffer which, along with the crosslinking degree, affected some hydrogel properties, including porosity and swelling rate. According to the results, crosslinking with 3 mg/ml of L-cysteine achieved the best hydrogels with regard to drug release. The release studies were performed using non-crosslinked and crosslinked hydrogels. In all hydrogel samples, a slight initial burst release was observed for 6 hours. In crosslinked hydrogels, the paclitaxel release rate was more controlled compared to non-crosslinked samples and was faster in acetate buffer than in PBS, possibly due to higher crosslinking density and lower swelling capacity and porosity in PBS. Therefore, the viability of breast cancer cells was reduced slightly more when treated with the hydrogels prepared in acetate buffer. When hydrogels were exposed to high glutathione concentrations, approximately all of their paclitaxel content was released within 72 hours, which proved that the hydrogels possessed reduced stimuli responsiveness due to crosslinking (Nieto et al., 2022[83]).

Synthetic polymers can be considered an attractive alternative for developing hydrogels since they can be molecularly tailored by molecular weights, block structures, mechanical strength, as well as biodegradability (Zhu and Marchant, 2011[151]).

**Figure 6:**
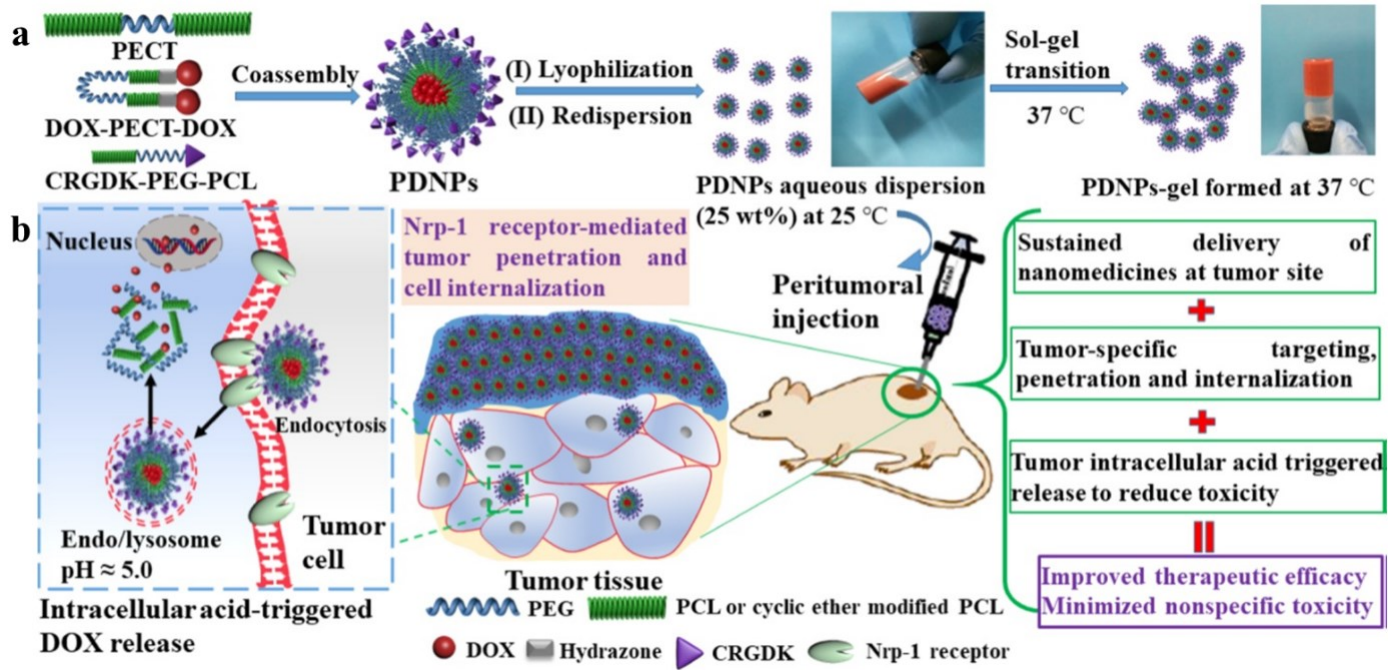
(a) Fabrication steps of doxorubicin-loaded prodrug nanoparticles (PDNPs) with the ability to undergo sol-gel transition upon injection to mice and forming a hydrogel depot. (b) The hydrogels release PDNPs, which degrade under acidic conditions in the cancer tissue environment and release doxorubicin. This method enhances the efficacy and reduces the toxicity of the treatment. Reprinted from Liu et al. (2019) copyright (2019) with permission from the American Chemical Society.

PEG, PLGA (Chang et al., 2011[21]), and poly(N-isopropyl acrylamide) (PNIPAAm) (Fong et al., 2017[31]) are some examples of popular synthetic polymers utilized to prepare hydrogels intended for localized delivery of anticancer drugs. PEG is the most extensively studied synthetic polymer used to make hydrogels because of its unique features like solubility in various solvents, non-immunogenicity, and non-toxicity (Kolate et al., 2014[59]). Moreover, the terminal hydroxyl groups of PEG can be modified by numerous functional groups, such as thiol, acrylate, and carboxyl, or attached to other molecules. PEG-based hydrogels can be developed via free radical polymerization of PEG macromers or radiation crosslinking of PEG (Zhu, 2010[150]). For example, Liu et al. designed tumor-specific, doxorubicin-loaded prodrug nanoparticles self-aggregated hydrogel using PEG with M_n_ = 1500 Da to enhance tumor cell targeting and penetration for effective post-surgical prevention of breast cancer recurrence (Figure 6(Fig. 6); Reference in Figure 6: Liu et al., 2019[70]). The system was developed using the aqueous solutions of lyophilized pH-responsive powder of targeted doxorubicin-loaded prodrug nanoparticles. These carriers were formed using a modular co-assembly of a thermosensitive amphiphilic copolymer, acid-cleavable PEGylated polymeric doxorubicin prodrug, and tumor-specific targeting peptide (CRGDK). This study indicated that a single administration of prodrug nanoparticles can develop a long-acting depot to release the nanocarriers in the tumor site for over three weeks and provide an obvious effect against tumor recurrence (Liu et al., 2019[70]). In a different study, thermosensitive PLGA-PEG-PLGA hydrogels were prepared for treating postoperative glioblastoma recurrence. A gene complex was formed with a non-viral vector (i.e., G5-BGG) and shRNA plasmid, and the complex was then loaded in the hydrogels. The hydrogels were formed due to a temperature shift by locally injecting the PLGA-PEG-PLGA solution and the gene complex inside the postoperative cavity. The gene complex showed a sustained release behavior due to the slow hydrogel degradation. According to the results, 60 % of the gene complex was released in 2 days, while about 80 % was released within one week. According to the *in vivo* results, combining the hydrogels with temozolomide could effectively enhance the permeation of macrophages into the tumors and increase the animal survival time. Furthermore, it could downregulate the expression of CD47 proteins, which increases the phagocytosis of cancer cells (Song et al., 2021[105]).

Natural/synthetic hybrid hydrogels can be developed by combining natural and synthetic polymer blocks generally through polymerization or chemical conjugation. The nature and size of these building blocks at the molecular level dictate the hybridization process. The hybrid hydrogels with functional blocks could be developed with desirable mechanical and physical characteristics, tunable kinetics, and stimuli-responsiveness for targeted drug delivery (Palmese et al., 2019[86]; Vasile et al., 2020[122]). Therefore, hybrid hydrogels have become the focus of major interest in cancer therapy (Fong et al., 2017[31]; Pantshwa et al., 2018[88]). Recent work by Chen's group (Fong et al., 2017[31]) has employed an *in situ* forming thermo-sensitive hybrid hydrogel (HA-CS-g-PNIPAAm) to deliver doxorubicin-loaded, folic acid-conjugated graphene oxide nanocarriers for breast cancer therapy. The hydrogel undergoes sol-gel transition after body injection and turns into a gel depot for drug delivery, which avoids surgery-assisted implantation, facilitates the modification of drug release rate by remodeling the formulation, and improves body excretion after achieving the intended purposes. Graphene oxide is considered to be generally safe for administration *in vivo*. Furthermore, PNIPAAm, which makes the hydrogels thermoresponsive, is a non-biodegradable polymer. To produce practicable hydrogels, PNIPAAm was grafted with other biocompatible materials to increase the safety and mechanical properties of the hydrogels. It has also been reported that PNIPAAm with low molecular weight exhibits better biocompatibility by excretion through renal clearance. Therefore, PNIPAAm with a molecular weight of 22 kDa was used in this study and histological analysis revealed that the hydrogels did not alter the renal and hepatic function significantly. The nanocarrier-embedded hydrogel provided controlled and site-specific delivery of doxorubicin via slow degradation of the hydrogel (~ three weeks) and subsequent cellular uptake of released nanocarriers through their interactions with folate receptors on the cancer cells. The particles indicated a pH-triggered release profile with ~5 times higher drug released at endosomal pH (~ 5.5) than at physiological pH (7.4), while the release behavior of the nanocomposite hydrogel displayed the same pH dependence as the nanocarriers but was much slower (Fong et al., 2017[31]). 

Similarly, some other researchers have also focused on the incorporation of nanoparticles within the hydrogel network to reinforce the structure of polymeric hydrogels and to provide multiple functionalities. Until now, a wide variety of nanoparticles, such as lipid-based, polymeric, and metallic ones have been integrated within the networks of hydrogels to develop nanocomposite hydrogels with superior features and tailored functionality for more efficient cancer treatment (Table 3(Tab. 3)). Incorporating nanoparticles inside hydrogels shows the potential to overcome the limitations in the administration of nanoparticles. The advantages of this method include reducing nanoparticle aggregation due to the high mechanical strength of hydrogels, enabling prolonged and controlled release of nanoparticles, stabilizing the nanoparticles inside the hydrogels, and maintaining them at the target site (Karimi et al., 2023[54]; Thoniyot et al., 2015[117]).

Taken together, the findings of studies on the hydrogels imply that the drug release behavior of these systems can be a function of both physicochemical properties of the cargoes and key characteristics of the hydrogels, such as network structure, hydrophilicity, crosslinking density, and degree of ionization of functional groups (Jaiswal et al., 2013[50]; Li and Mooney, 2016[68]; Zhuang et al., 2020[152]). These features can be exploited to provide controlled drug delivery for periods lasting from a few hours to several weeks. The drug release rates of the articles discussed in this section varied from 90 % in 12 hours due to water-solubility of drug molecules (Li et al., 2020[66]), to 68 % within 30 days (Jaiswal et al., 2013[50]) which was discussed in detail. Different mechanisms are responsible for drug release from the hydrogels, including diffusion (e.g., when the size of the drug is smaller than the hydrogel mesh size), swelling, and chemical mechanisms. When the mesh size of hydrogel is much smaller than the drug molecule, the drug is physically entrapped within the hydrogel by strong steric hindrance and could be released in a controlled manner upon degradation of the hydrogel network or expansion of the mesh size in response to stimuli, such as changes in pH, temperature, or ionic strength. Furthermore, the release pattern could also be controlled in physical hydrogels by engineering biodegradation in physiological conditions, mediated by hydrolysis, enzymes, or erosion (Andrade del Olmo et al., 2022[9]). 

## Wafers/Sponges

Lyophilized wafers or sponges are among novel drug delivery systems, that can be described as gas dispersions within solid matrices with interconnected pores (Ng, 2020[82]). They are generally developed by freeze-drying of polymeric solutions or gels, and thus possess a porous structure because of the removal of ice crystals by the lyophilization process (Ayensu et al., 2012[11]; Boateng et al., 2010[16]; Vaezi et al., 2022[121]). Freeze-drying is crucial for maintaining the texture and other features of the product (Ng, 2020[82]). 3D printing, as explained in previous sections, is a novel technology that is also applicable for fabricating wafers to increase patient adherence and medicine personalization. Different 3D printing techniques, such as extrusion-based 3D fabrication have been utilized for preparing pharmaceutical products (Abdelkader et al., 2021[1]). 

Wafers usually bear high drug loading capacity, mainly related to their porous nature and large surface area (Boateng et al., 2010[16]). In addition, they maintain their swollen state for a long period, allowing for long residence time and effective absorption of therapeutics (Ayensu et al., 2012[11]). With such potential advantages, different wafers for cancer therapy have recently been designed using natural polymers like silk fibroin (Yavuz et al., 2018[139]), gelatin, and chitosan (Zhang et al., 2018[146]) and synthetic polymers, such as poly[1,3-bis(p-carboxyphenoxy) propane-co-sebacic acid] (p(CPP:SA); 80:20 molar ratio) (Duntze et al., 2013[28]) (Table 4(Tab. 4); References in Table 4: Aravind et al., 2021[10]; Chaichana et al., 2011[20]; De Bonis et al., 2012[26]; Duntze et al., 2013[28]; Hayashi et al., 2020[39]; Sun et al., 2013[111]; Wang et al., 2021[124]; Yavuz et al., 2018[139]; Zhang et al., 2018[146]).

**Figure 7:**
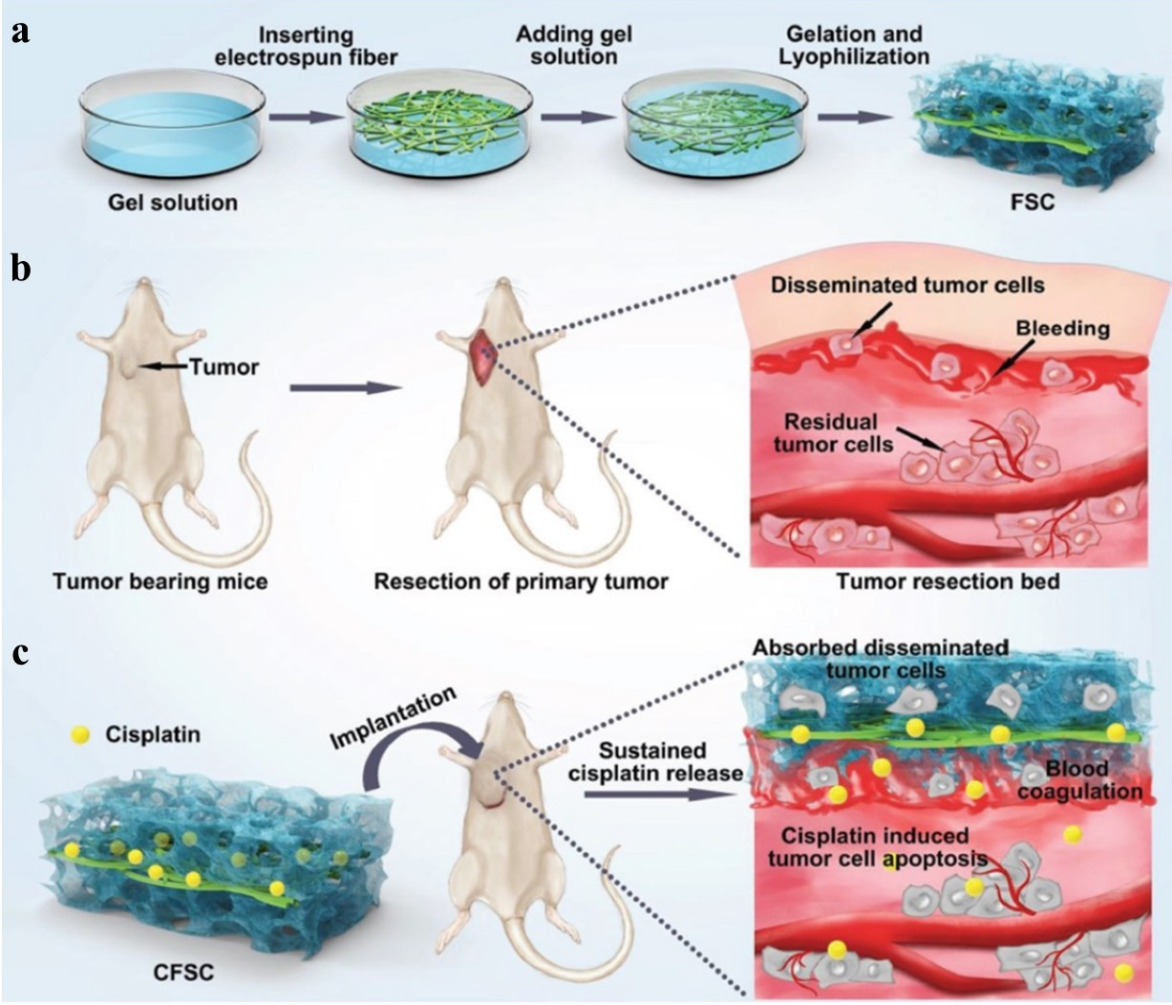
Preparation and delivery of sandwich-like composites of fibers and sponge loaded with cisplatin for preventing postoperative tumor recurrence. (a) To prepare the composites, electrospun fibers were inserted into the gel solution and lyophilized after forming a gel. (b) A postoperative cancer model was prepared using mice. The tumor resection bed is illustrated. (c) The drug-loaded composites were implanted in the tumor bed, which induced blood coagulation and residual tumor cell apoptosis. Reprinted from Zhang et al., 2018b with permission from John Wiley and Sons.

As an example of this type of implant, a sandwich-like cisplatin-loaded fibers/sponge composite was designed to combine hemostasis and chemotherapy, aiming to inhibit recurrence and metastasis following the resection of primary tumors (Figure 7(Fig. 7)) (Zhang et al., 2018[146]). The polymers used to develop the sponge were chitosan and gelatin, which were chosen for their ability to activate coagulation via strong hemaglutination and improvement of platelet aggregation, respectively. The results of this study showed that the composite not only efficiently stopped the bleeding and adsorbed disseminated cancer cells after tumoral resection but also released cisplatin in a sustained manner (~ 16 % release in PBS (pH = 7.4) after 5 days) to kill residual cancer cells as well as those concentrated within the composite, leading to improved anti-recurrence and anti-metastasis efficacy (Zhang et al., 2018[146]).

In an *in vitro* study, doxorubicin-loaded collagen sponges with alternating magnetic field (AMF)-controlled drug release properties were prepared. The incorporated magnetic iron oxide nanoparticles could generate heat upon exposure to AMF, which leads to a remote-controlled release pattern by on and off switching of AMF. According to the results, thermally crosslinking the sponges for 6 hours completely prevented the undesired drug release in PBS at 37 °C, without applying AMF, but enabled AMF-controlled drug release. Moreover, using thermal treatment combined with doxorubicin release exhibited more efficient tumor cell-killing properties compared to thermal treatment alone, and the effects lingered on after terminating the AMF exposure (Hayashi et al., 2020[39]). 

The Gliadel^®^ wafer is possibly the most successful implantable delivery system for treating high-grade malignant glioma. Developed by Langer and Brem in the 1980s, this implant was approved by FDA in 1996 (Abdelkader et al., 2021[1]; Wolinsky et al., 2012[128]) and has been investigated from its chemistry to its performance in various clinical trials (Chaichana et al., 2011[20]; De Bonis et al., 2012[26]; Duntze et al., 2013[28]). This system is composed of a copolymer, p(CPP:SA), which is dissolved with the chemotherapeutic carmustine in an organic solvent, spray-dried into microparticles, and finally, compression molded into the wafer (Wolinsky et al., 2012[128]). Gliadel^®^ wafers have shown carmustine release within rat brains for a time period of about 5 days followed by slow degradation of their polymeric matrix at 6 to 8 weeks post-implantation (Fleming and Saltzman, 2002[30]). Based on the clinical findings, using Gliadel^®^ together with surgical intervention and, in most cases, radiotherapy patients with newly diagnosed, malignant glioma showed an enhanced survival from 11.6 months to 13.8 months. Moreover, median survival increased about two months for cases treated following surgery for recurrent disease (Wolinsky et al., 2012[128]).

In this section, the behaviors of release for different cargoes in relation to system design, polymer swelling capacity, and matrix erosion are explained. But, in general, wafers are designed to provide sustained release of their loaded chemotherapeutics over sufficiently long periods, varying from several hours (Hayashi et al., 2020[39]) to months (Aravind et al., 2021[10]), allowing achievement of prolonged therapeutic concentrations at tumor sites. Different drug release mechanisms are present in wafers. For instance, in a study conducted by Sun et al., sponge degradation led to sufficient water penetration and formation of diffusion channels for drug release. The drug release rate remained relatively constant using erosion and diffusion release mechanisms (Sun et al., 2013[111]). Another important factor affecting controlled drug release in wafers is the interactions of the drug molecule with the crosslinkers (Ng, 2020[82]) or with the polymer (Zhang et al., 2018[146]). As explained before, wafers could also initiate drug release in response to external stimuli (e.g., AMF exposure) (Hayashi et al., 2020[39]). Overall, wafers could provide a controlled and sustained release of different drugs and could easily be upscaled to industrial production due to its relatively facile preparation methods compared to other novel drug delivery systems (e.g., nano-systems) (Ng, 2020[82]).

## Osmotic Pumps

Osmotic pumps were first devised in the 1950s by Australian pharmacologists, Rose and Nelson, for the delivery of drugs. Since then, different designs have found potential clinical use for treating a variety of diseases (Kumar and Pillai, 2018[62]). They are conventionally composed of a drug reservoir, an osmotic agent, and a semipermeable membrane. The most commonly used semipermeable membrane is cellulose acetate with various acetyl contents. Lactose, fructose, mannitol, sodium chloride, and some of their mixtures are available osmotic agents in the market (Almoshari, 2022[4]). Upon implantation of these systems, water molecules are drawn via the semipermeable membrane due to an osmotic pressure difference between the moisture of the surrounding interstitial fluid and the osmotic agent. Consequently, expansion of the osmotic agent pushes the piston forward and thereby expels the drug molecules from the reservoir through an orifice (Pons-Faudoa et al., 2019[95]). A nearly constant (zero-order) drug release rate is maintained in some studies (Gong et al., 2015[34]; Pan et al., 2017[87]). Many factors could affect the design of osmotic pumps, including the types of semipermeable membrane, polymer, and plasticizer, drug solubility, and osmotic pressure. Apart from providing sustained drug release, these systems possess many other advantages, such as easy preparation and handling and high correlation between* in vitro* drug dissolution and* in vivo* bioavailability. Furthermore, the drug release is controlled by osmotic pressure and therefore is independent of different physiological factors such as pH (Almoshari, 2022[4]).

The Alzet® osmotic pump is today probably the most well-known example of osmotic pumps, which is capable of releasing its drug content at controlled rates for durations ranging from 1 d to 6 weeks (Herrlich et al., 2012[41]; Kumar and Pillai, 2018[62]). It was fabricated for research purposes and is commercially available (DURECT Corporation, Cupertino, CA, USA). This pump is cylindrically shaped and comprises a collapsible core reservoir composed of an impermeable thermoplastic hydrocarbon elastomer that is enwrapped by an osmotic driving agent layer. A semi-permeable membrane of a cellulose ester blend covers the external surface of the pump. The pressure developed by water entering displaces the stored drug volume (Herrlich et al., 2012[41]). The Alzet® pump could be implanted in numerous animal species and in a variety of anatomical locations. By providing continuous delivery of anti-cancers, numerous therapeutic regimes can be applicable to the treatment of various malignancies that have been investigated using this pump (Biddlestone-Thorpe et al., 2013[15]; Herrlich et al., 2012[41]; Mao et al., 2015[77]; Yu et al., 2014[141]) (Table 5(Tab. 5); References in Table 5: Altanerova et al., 2012[7]; Biddlestone-Thorpe et al., 2013[15]; Kim et al., 2012[58]; Mao et al., 2015[77]; Miyake et al., 2014[79]; Qorri et al., 2022[98]; Tibensky et al., 2022[119]; Yang et al., 2016[138]; Yu et al., 2014[141]). The rate of drug release in Alzet® pump depends on the drug concentration and the volume of water entering the semipermeable membrane. By altering the permeation of semipermeable membranes, the release rate could be controlled (Almoshari, 2022[4]).

In a study by Lun-Quan Sun's team, an active DNAzyme targeting the bcl-xL gene was delivered to PC3 prostate tumor-bearing mice via an Alzet^®^ pump at a dose rate of 12.5 mg/kg/d for 2 weeks. By achieving an accurate delivery of the DNAzyme over the study period, a significant chemosensitization with the DNAzyme was achieved for taxol treatment (Yu et al., 2014[141]). Alzet^®^ pump is a non-biodegradable system (Wright, 2010[130]). However, biodegradable micro-fabricated osmotic pumps have been developed by using biodegradable materials for the controlled release of basic fibroblast growth factor (Ryu et al., 2007[101]).

## Conclusion and Future Perspectives

During the past decades, tremendous efforts have been devoted to the development and characterization of versatile IDDS to both increase the effectiveness of chemotherapeutics and decrease their systemic toxicity, and notable progress has been achieved, as reflected by a growing number of publications. Although utilization of these vehicles has promising potential in cancer therapy, there are knowledge gaps regarding the most effective type of formulations for prolonged local delivery of anti-cancer agents. To address this need, we have discussed the formulation and application of different IDDS in the targeted delivery of chemotherapeutic agents by reviewing the representative literature. The preparation strategies of the IDDS including novel techniques of 3D printing and microfluidic, and their drug release profiles have also been outlined in this review article. Furthermore, we demonstrated the effectiveness of IDDS in treating various types of cancer, including breast, liver, and lung cancer, which are among the most prevalent cancer types worldwide. The main preclinical and clinical achievements of these systems are provided in Tables 1-5(Tab. 1)(Tab. 2)(Tab. 3)(Tab. 4)(Tab. 5). The purpose of this study was to provide a comprehensive review of IDDS to better understand their role in cancer treatment and help researchers develop new therapeutic strategies.

Many interesting enhancement opportunities could be available for IDDS in the future. For instance, advanced features including stimuli-sensitive drug release, remotely controllable and programmable features, external drug refill, non-invasive implantation procedures, and self-administration feasibility of IDDS could be added to the systems (Kar et al., 2022[53]). Moreover, novel approaches, including 3D printing and microfluidic techniques (Zhang and Khademhosseini, 2017[145]), micro-/nano-electro-mechanical systems (MEMS/NEMS), which enable the opportunity to fabricate complicated and miniaturized structures and personalized devices, could be utilized more extensively. 

Although IDDS have achieved many developments, there are still several challenges to address. There is a noticeable need for more well-designed clinical trials with appropriate methodology, number of participants, treatment duration, and follow-up sessions to determine the safety and efficacy of the treatments. Concerns regarding various inflammatory responses attributed to the local accumulation of chemotherapeutics and polymeric substances (Taraballi et al., 2018[116]), impairment of the natural tissue repair process after surgical intervention (Onuki et al., 2008[85]), formation of a thick collagenous fibrous capsule around IDDS (Dolan et al., 2019[27]) that acts as a drug release barrier, large-scale production, and high production yield should be adequately addressed before clinical applications. Ideal IDDS for cancer therapy should have tissue repair promotion, inflammation management, and tumor suppression properties, simultaneously. 

## Notes

Maryam Ebrahimnia and Sonia Alavi contributed equally as first author.

## Declaration

### Funding

This work was financially supported by Shahid Beheshti Medical University. 

### Competing interests

There are no financial or non-financial interests to declare.

### Author contributions

All authors contributed to the literature search, data analysis, project administration, and writing the original draft of the manuscript. Conceptualization and methodology were performed by Maryam Ebrahimnia, Sonia Alavi, and Azadeh Haeri. Editing and revision of the manuscript and project supervision was performed by Azadeh Haeri. All authors read and approved the final manuscript.

### Data availability statement

All data are described in the article.

### Ethical standards

Not applicable.

## Figures and Tables

**Table 1:**
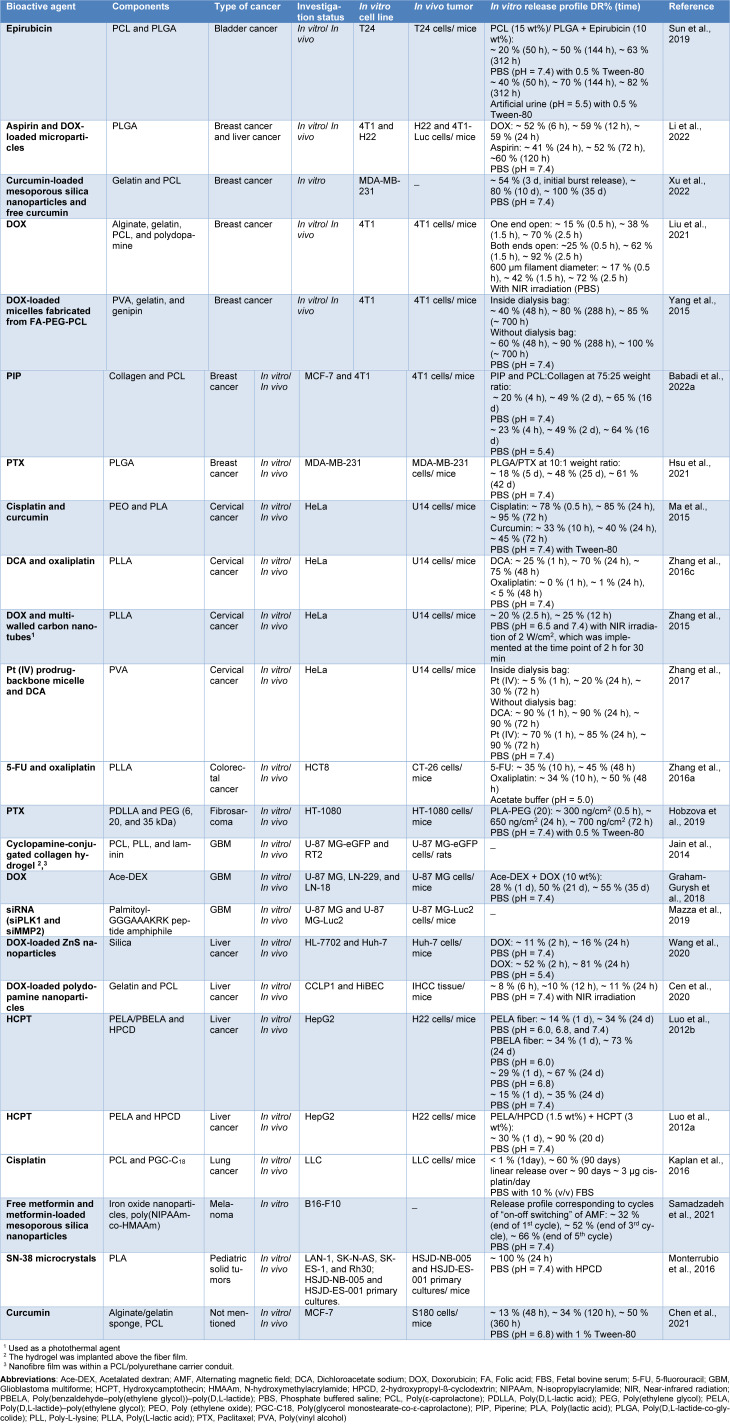
Studies on implantable nanofiber systems for localized anti-cancer drug delivery

**Table 2:**
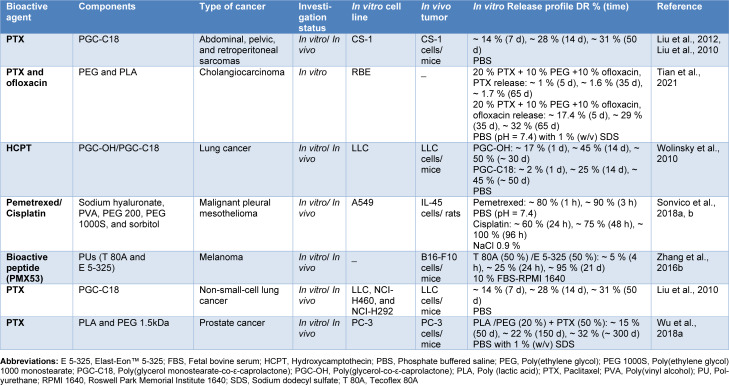
Studies on implantable films for localized anti-cancer drug delivery

**Table 3:**
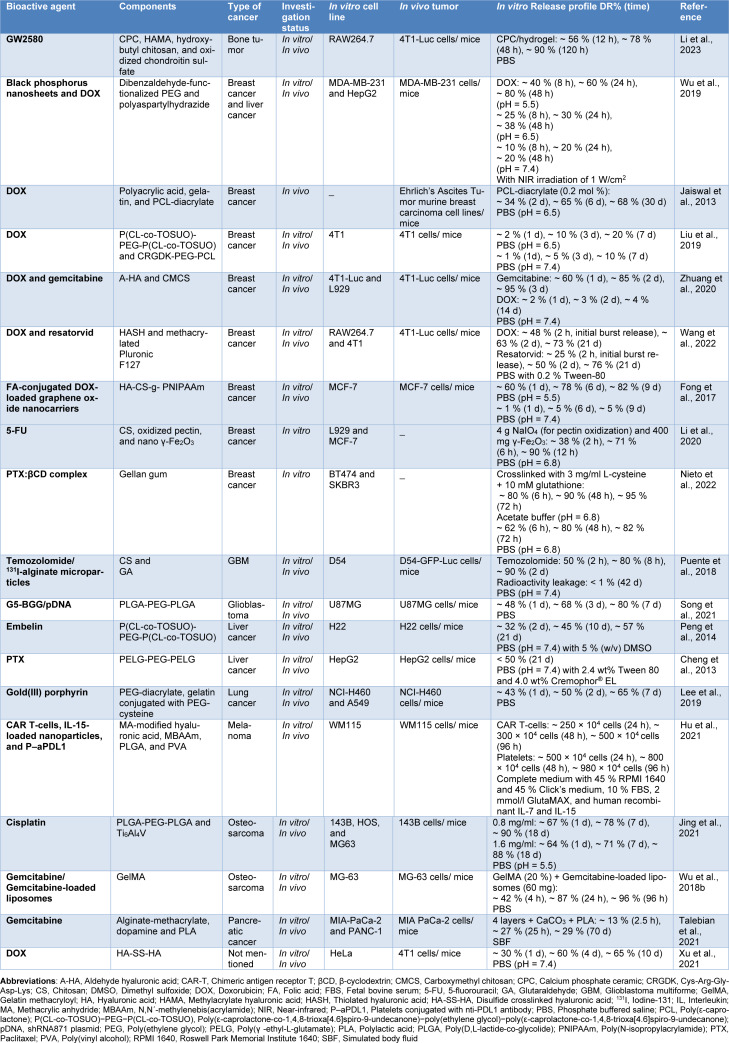
Studies on implantable hydrogel systems for localized anti-cancer drug delivery

**Table 4:**
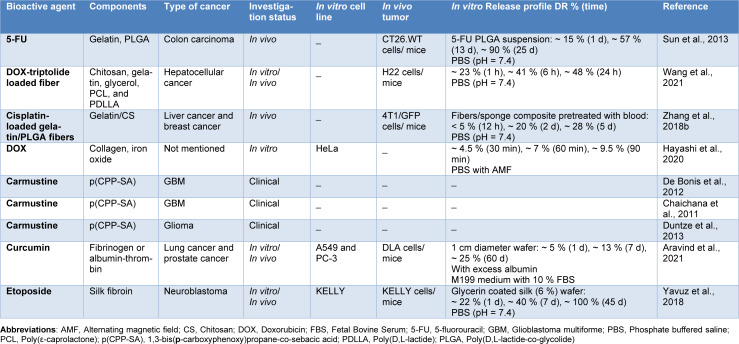
Studies on implantable wafers and sponges for localized anti-cancer drug delivery

**Table 5:**
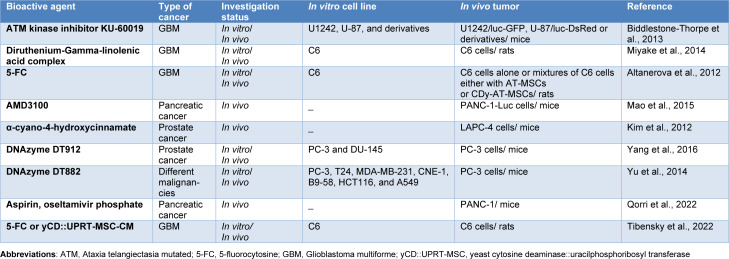
Studies on implantable ALZET^®^ osmotic pump for localized anti-cancer drug delivery

**Figure 1:**
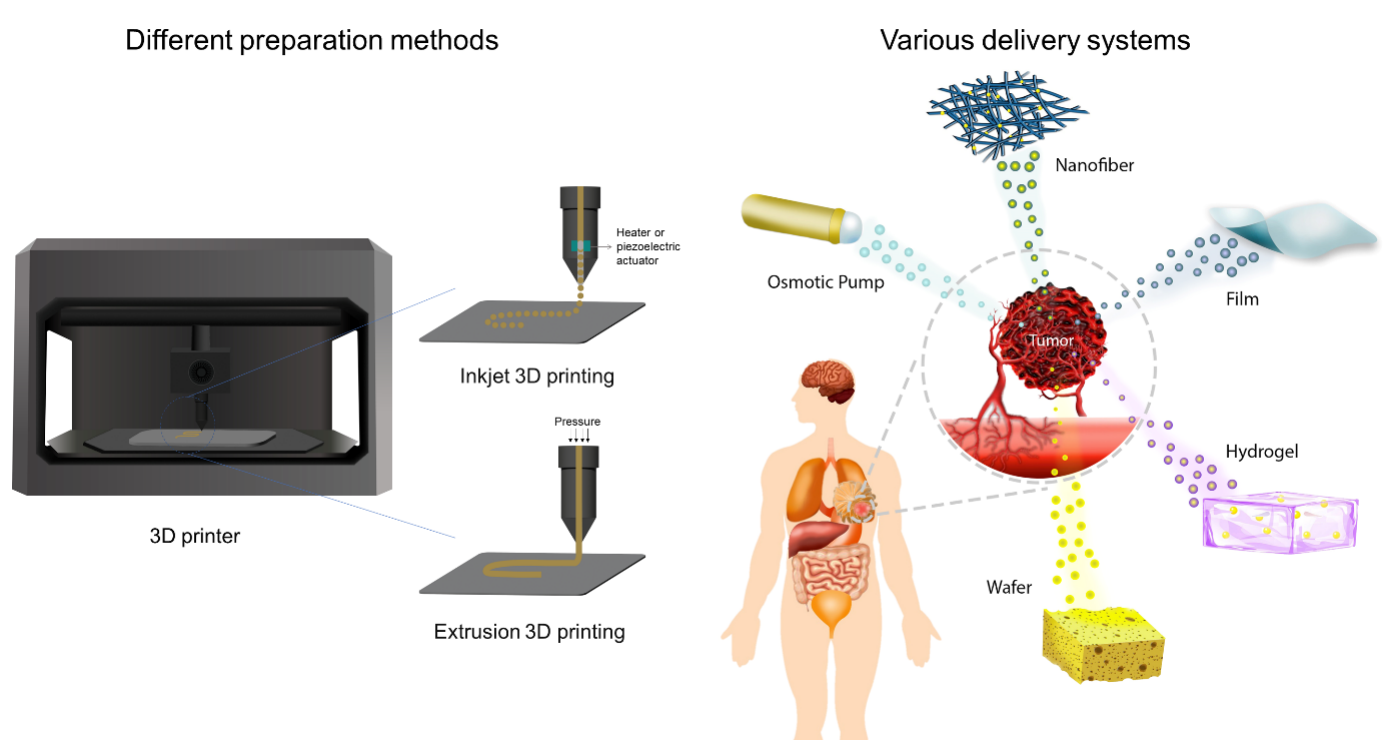
Graphical abstract
